# Cross-cultural adaptation and validation of the Italian versions of the Kujala, Larsen, Lysholm and Fulkerson scores in patients with patellofemoral disorders

**DOI:** 10.1186/s10195-018-0508-9

**Published:** 2018-09-12

**Authors:** Simone Cerciello, Katia Corona, Brent Joseph Morris, Enrico Visonà, Giulio Maccauro, Nicola Maffulli, Mario Ronga

**Affiliations:** 1 Casa di Cura Villa Betania, Rome, Italy; 2 Marrelli Hospital, Crotone, Italy; 3 Department of Medicine and Health Sciences “Vincenzo Tiberio”, University of Molise, Campobasso, Italy; 4 Shoulder and Elbow Surgery, Fondren Orthopedic Group, Texas Orthopedic Hospital, Houson, Texas, USA; 5 Ospedale di Este, ULSS 17, Este, Italy; 6 Università Cattolica del Sacro Cuore, Rome, Italy; 7 Department of Musculoskeletal Disorders, School of Medicine and Surgery, University of Salerno, Salerno, Italy; 8 Centre for Sports and Exercise Medicine, Queen Mary University of London, Barts and The London School of Medicine and Dentistry, London, UK

**Keywords:** patellofemoral pathology, cross-cultural adaptation, validation, translation

## Abstract

**Background:**

The Kujala, Fulkerson, Larsen and Lysholm questionnaires have been demonstrated to be reliable and sensitive in assessing patients with patellofemoral pathology. The purpose of this study is to translate and cross-culturally adapt into Italian the English versions of the Kujala, Fulkerson, Larsen and Lysholm questionnaires, and undertake reliability and validity evaluations of the Italian versions of these scores in patients with patellofemoral pathology.

**Materials and methods:**

The cross-cultural adaptation process was carried out following the simplified Guillemin criteria. The questionnaires were administered to 63 patients with either patellar instability or painful patella syndrome. To assess the validity of the questionnaires, they were compared with the Oxford knee score. The questionnaires were administered to a subsample of 33 patients 5 days later to assess test–retest reliability.

**Results:**

The interclass coefficient correlation was 0.96 for the Kujala score, 0.92 for the Larsen score, 0.96 for the Lysholm score, 0.94 for the Fulkerson score (*P* < 0.01), and 0.83 for the Oxford score. Pearson’s correlation was0.96 between the Kujala and Oxford scores, 0.90 between the Larsen and Oxford scores, 0.94 between the Lysholm and Oxford score, and 0.93 between the Fulkerson and Oxford scores. Responsiveness, calculated by standardized response mean, was 1.2, and effect size was 1.4.

**Conclusions:**

The Italian versions of the Kujala, Larsen, Lysholm and Fulkerson scoring systems were shown to be equivalent to their English versions and demonstrated good validity, reliability and responsiveness to surgical treatment of patellofemoral pathology. To the best of the authors’ knowledge, this is the first attempt to adapt four of the most common patellofemoral-specific scoring scales to the Italian language.

**Level of evidence:**

Level II.

**Electronic supplementary material:**

The online version of this article (10.1186/s10195-018-0508-9) contains supplementary material, which is available to authorized users.

## Introduction

Patellofemoral pathology is frequently encountered in orthopaedic practice and commonly affects both young and old patients. The correct diagnosis of patellofemoral pathology may be difficult to ascertain, especially in younger patients, since the pathogenesis is frequently multifactorial and precise classification is often not possible. Patient history and physical examination are key for correct diagnosis and proper treatment. There are many options for conservative and surgical management [3]. It is imperative to use a validated disease tool to evaluate the preoperative status and clinical outcome of these treatments. Objective imaging parameters have been proposed to assess the severity of patellofemoral pathology and the outcome of surgical procedures: patellar height, trochlear dysplasia, tibial tuberosity–trochlear groove distance and patellar tilt. Unfortunately, these objective measures do not always correlate with subjective patient outcome measurements.

Recently, great attention has been paid to patient-oriented measurement tools. Patient-reported outcome measurement tools are crucial to assess and quantify outcomes in orthopaedics [1, 11]. An appropriate patient-reported outcome measurement tool should include general health aspects and disease-specific measurements [6]. Several disease-specific questionnaires for knee and patellar pathology have been proposed in recent decades. The Fulkerson, Kujala, Larsen and Lysholm scoring systems are the most common and reliable patient-reported outcome tools for knee and patellofemoral pathology [4, 8, 10, 14]. These scoring systems are now widely used across several countries. It would be very desirable to translate, adapt and evaluate these scoring systems for non-English-speaking subjects. Unfortunately, simple translation is not sufficient to adapt these score sheets to different countries’ languages. Questionnaires must be validated through a complex process of translation and adaptation to be accepted as useful tools [5, 13]. Therefore, when a health-related quality of life (HRQOL) measure is introduced into a new country, two options are available, as described by Guillemin [5]. A new scoring system can be created, or a previously created and validated questionnaire can be adapted to a new country through a cross-cultural adaptation process. An adaptation to a new country by simple translation without consideration of language and cultural differences leads to a useless tool. A translation should consider the differences in cultural habits and language. A cross-cultural adaptation is necessary to adapt a single scoring system to a given country and language. Guillemin described the process of adaptation of these questionnaires to specific languages and their subsequent validation for a specific country. This process consists of translation of the questionnaire and subsequent adaptation to idioms, culture and lifestyle. He proposed guidelines for a five-step process, including translations and back-translations by qualified people, a committee review of these translations and back-translations, pre-testing for equivalence and finally a re-examination of the weighting of scores. These points are particularly relevant when considering orthopaedic scoring systems, which are often created for English-speaking people. Patients from other countries could consider their quality of life or subjective assessment in a different manner according to their comprehension of the questionnaire as well as cultural differences. Furthermore, statistical evaluation including reliability, sensitivity and responsiveness must be carried out. Scoring systems are considered to be effective tools after undergoing this rigorous assessment and are deemed suitable to be used in different countries [15].

The aim of our study is to translate and adapt the English Kujala, Fulkerson, Larsen and Lysholm questionnaires to Italian and to perform reliability and validity evaluations of the Italian versions of these scores in patients with patellofemoral pathology.

## Materials and methods

### Outcome tools

Several disease-specific measurement tools for patellofemoral pathology have been introduced in recent decades in English-speaking countries to evaluate the influence of knee pathology on daily living activities. These validated tools are reliable, easy to use, and have become common in orthopaedic practice. The Kujala score was introduced in 1993 for patients with patellofemoral pathologies. It is a 100-point scale consisting of 13 items with a score varying from 5 to 10 points per item [8]. A score of 0 points indicates the most severe limitation, whereas a score of 100 points indicates a normal situation.

The Lysholm questionnaire was initially proposed in 1982 in patients with knee ligament injuries. It is a 100-point scale and consists of eight items. Pain and instability are the most heavily weighted items (25 points each) [14]. The Fulkerson questionnaire was published in 1990 as an evolution of the Lysholm questionnaire to evaluate patellofemoral symptoms and results of anterior tibial tubercle transfer. It is a 100-point scoring system, and consists of seven items with variable points per item ranging from 0 to 45. The most heavily weighted item is pain with a maximum score of 45. This questionnaire has shown to be a useful tool in patients with patellofemoral problems and in patients with knee ligament instability [4].

The Larsen–Lauridsen score is a 20-point questionnaire that was introduced in 1982, following the previous questionnaires by Insall, Crosby and Heyewood. It is a subjective tool used in the evaluation and treatment of patellofemoral disorders. It consists of six items with values ranging from 2 to 4 points [10]. Similar to the Kujala score, both the Lysholm, Fulkerson and Larsen questionnaires attribute a score of 0 points to the most severe knee limitation and a maximum score to the normal situation. A process of cross-cultural adaptation of these questionnaires was performed. A translation was performed using the protocol of cross-cultural adaptation proposed by Guillemin [5]. For each of the previous score sheets, a forward translation was carried out by two independent physicians. These two translations were compared to create a new single one. Subsequently, a backward translation was performed by another physician and checked for inconsistencies with the original English text. Considering that no major cultural differences between Italians and Americans are present, no further adaptation process was performed. The final version of these four questionnaires was then administered to a selected population (see Additional file 1 for the Italian versions of the four questionnaires). Moreover, we wanted to assess the correlation between these score sheets and a previously validated subjective questionnaire, as a result, the Italian version of the Oxford knee score was also administered to all patients [12].

### Patients

Sixty-three patients (8 male, 55 female) were included in our study: 40 patients with a minimum of three documented episodes of patellar dislocation and 23 patients with patellofemoral pain syndrome. Inclusion criteria consisted of: sports practice at least at the recreational level before initial injury; unsuccessful intensive rehabilitation protocol before surgery for at least of 6 months; acceptance of the proposed surgical procedure and the consequent postop rehabilitation program. All of the patients with patellar dislocation were treated with medial patellofemoral ligament (MPFL) reconstruction, while the patients with patellofemoral pain syndrome were treated with resection of the lateral retinaculum of the knee. The mean age at time of evaluation was 29 (± 6.6) years. All patients were evaluated by physical examination and radiographic examination using anterior-posterior (AP), lateral and Merchant radiographs. All patients were asked to complete the Kujala, Fulkerson, Larsen, Lysholm and Oxford questionnaires independently and in presence of an orthopaedic resident. The time necessary to complete each one of the questionnaires as well as any difficulty in answering a single question was recorded. To reduce the risk of short-term clinical change, no treatment was provided to these patients over a 5-day interval. To perform test–retest evaluation, 33 patients were asked to complete the same questionnaires 5 days later, assuming that the clinical situation and severity of symptoms had not changed during this short interval.

### Psychometric properties and statistical analysis

Statistical analysis was performed using SPSS 24 for Windows. Data are reported as mean, median, standard deviation and interquartile range. The Shapiro–Wilk test was used to assess normality. Validity, reliability and responsiveness were investigated. All tests were two-sided, and value of *P* < 0.05 was considered to indicate statistical significance.

#### Feasibility

Feasibility was assessed by considering the time required to fill in the questionnaire, the ease of compilation and the proportion of incomplete questionnaires.

#### Internal consistency

Internal consistency designates the correlation between items that make up the score and is assessed using Cronbach’s alpha coefficient with 95 % confidence interval (CI). Values equal to or above 0.7 indicate acceptable reliability for scales which are used as research tools to compare groups. Cronbach’s alpha ranged from 0.78 to 0.97, indicating good homogeneity.

#### Construct validity

Construct validity compares the outcome measurement tool with a gold standard when no “true value” is available. Pearson’s correlation coefficient was used to correlate the preoperative measurement and the changes of score for the Kujala, Fulkerson, Larsen, Lysholm and Oxford questionnaires.

#### Test–retest reliability

Reliability is the concept that repeated administration of a measurement tool will yield the same result in stable subjects. Participants were asked to complete the Kujala, Fulkerson, Larsen, Lysholm and Oxford questionnaires, then again 5 days later, to assess test–retest reliability, expressed as the intraclass correlation coefficient (ICC) with 95 % CI. An ICC of more than 0.80 is considered an indicator of good reliability. Absolute reliability was determined by estimating the standard error of measurement SEM = (SDdiff √1 − ICC) and the minimal detectable difference MDD = 1.96 × √2 × SEM. A Bland–Altman plot shows the mean difference in test and retest values of the Kujala, Fulkerson, Larsen and Lysholm against the mean of these two measures.

#### Responsiveness

Responsiveness refers the ability of a questionnaire to reflect significant clinical change in the subject’s state and was assessed by the standardized response mean (SRM) and the effect size (ES). SRM is calculated as the difference between the preoperative and postoperative mean score, divided by the standard deviation (SD) of the difference. ES is calculated as the difference between the postoperative and preoperative mean score, divided by the preoperative SD.

#### Discriminant ability

Floor and ceiling effects are defined, respectively, as the percentage with the worst and best possible score of the total number of patients who compiled the questionnaire.

## Results

The average Kujala score was 70.59 ± 23.45 (median 70.59, range 18–100), the average Larsen score was 14.50 ± 3.88 (median 15, range 6–20), the average Lysholm score was 69.64 ± 26.62 (median 77, range 12–100), and the average Fulkerson score was 72.84 ± 25.88 (median 80, range 14–100). The Oxford knee score, which was chosen as gold standard, had an average value of 35.59 ± 10.43 (median 37, range 11–48).

### Feasibility

All four questionnaires (Kujala, Fulkerson, Larsen and Lysholm) were easily completed by all patients, and no difficulties were noted for any specific questions. The average time necessary to complete each of the four questionnaires was less than 10 min in all cases. All patients found the questionnaires to be relevant to their physical situation. Moreover, the mentioned items were extremely simple, and the back-translation had a high correspondence to the original version.

#### Internal consistency

Cronbach’s alpha was 0.930, 0.910, 0.920 and 0.910 for the Kujala, Fulkerson, Larsen and Lysholm score, respectively, indicating good homogeneity.

#### Construct validity

A correlation was performed to assess the construct validity between the Kujala, Fulkerson, Larsen and Lysholm scores with respect to the Oxford score. Since the Shapiro–Wilk test showed that the data distribution was normal (*P* > 0.05), Pearson’s was used. The correlation was 0.96 between the Kujala and Oxford scores, 0.90 between the Larsen and Oxford scores, 0.94 between the Lysholm and Oxford scores, and 0.93 between the Fulkerson and Oxford scores (Table 1). There were no floor and ceiling effects preoperatively or postoperatively for the total of the four questionnaires.

**Table 1:** Pearson’s correlation

	Oxford
Kujala	0.960**
Larsen	0.897**
Lysholm	0.938**
Fulkerson	0.926**

**Correlation significant at 0.01 level (two-tailed)

### Test–retest reliability

**Fig. 1:**
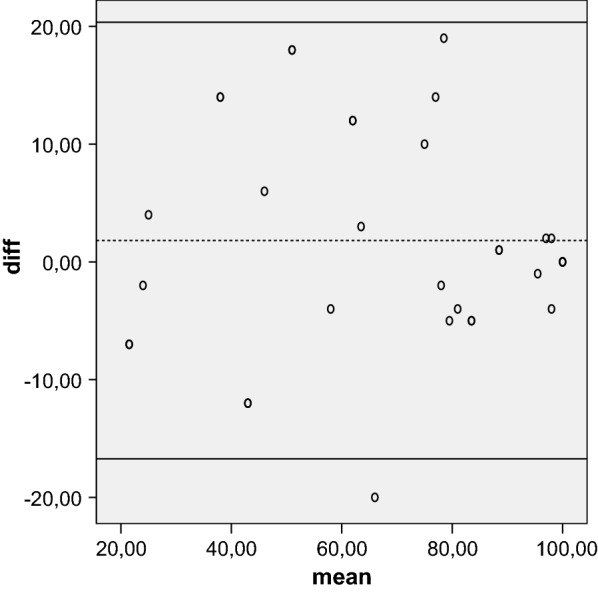
Bland–Altman plot showing test–retest results for 33 patients completing the Italian version of the Kujala index. The dashed line shows the mean difference; solid lines show the 95 % confidence interval

**Fig. 2:**
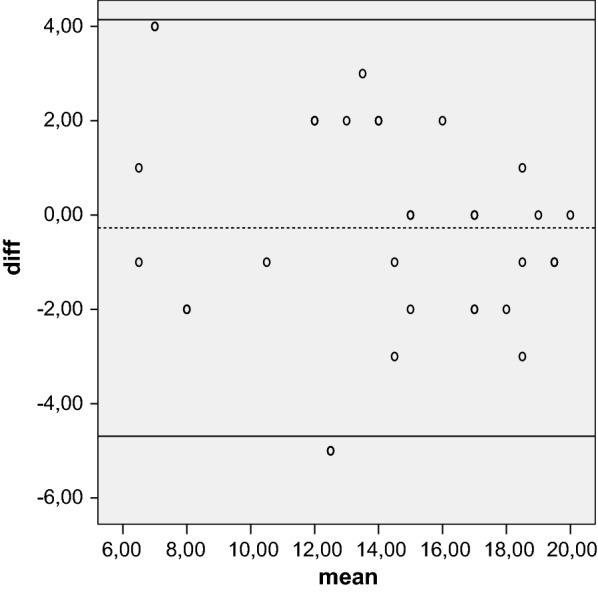
Bland–Altman plot showing test–retest results for 33 patients completing the Italian version of the Larsen index. The dashed line shows the mean difference; solid lines show the 95 % confidence interval

**Fig. 3:**
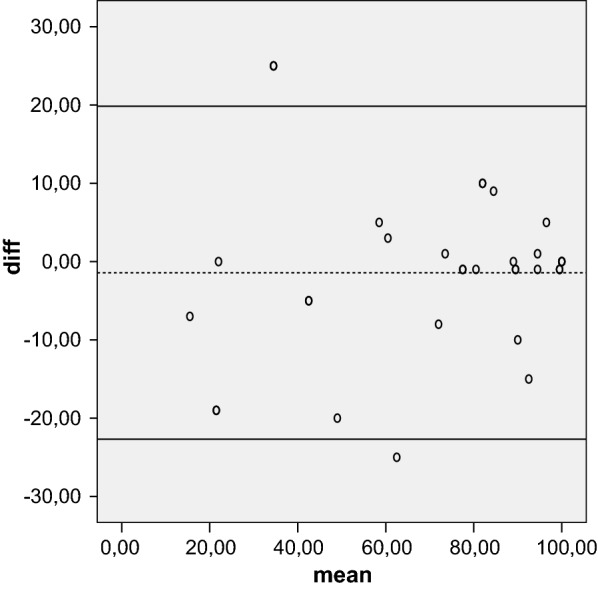
Bland–Altman plot showing test–retest results for 33 patients completing the Italian version of the Lysholm index. The dashed line shows the mean difference; solid lines show the 95 % confidence interval

**Fig. 4:**
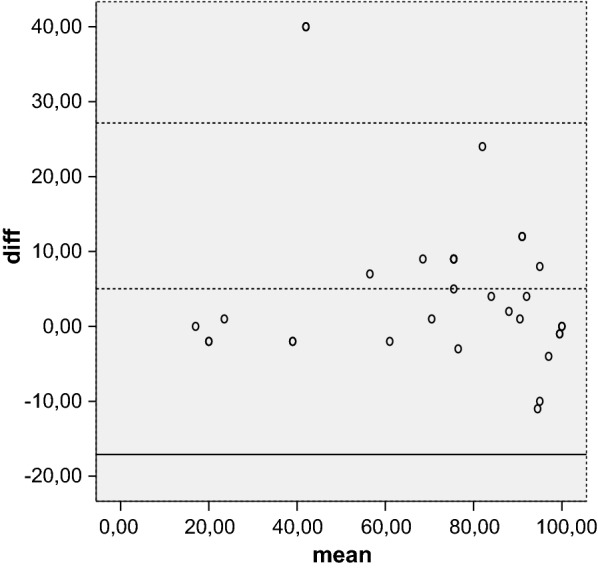
Bland–Altman plot showing test–retest results for 33 patients completing the Italian version of the Fulkerson index. The dashed line shows the mean difference; solid lines show the 95 % confidence interval

**Fig. 5:**
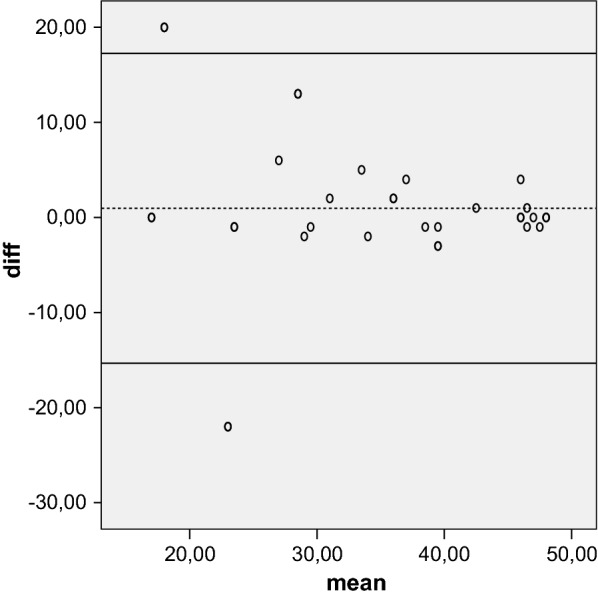
Bland–Altman plot showing test–retest results for 33 patients completing the Italian version of the Oxford index. The dashed line shows the mean difference; solid lines show the 95 % confidence interval

The mean of the five tests were calculated at initial evaluation and over a 5-day interval. Test–retest reliability showed that the results were highly reproducible over time. The ICC was 0.96 for the Kujala score, 0.92 for the Larsen score, 0.96 for the Lysholm score, 0.94 for the Fulkerson score, and 0.83 for the Oxford score. All values were highly statistical significant (*P* < 0.001). The SEM/minimal detectable change (MDC) was 0.83/2.64 for Oxford, indicating a smaller amount of measurement error in the screen. The Bland–Altman plots showed small mean difference (Figs. 1, 2, 3, 4, 5).

#### Responsiveness

All four questionnaires were responsive and sensitive to detect clinical changes in the study population over a 12-month period following surgical treatment. The SRM was 1.3 for the Kujala score, 0.9 for the Fulkerson score, 1.1 for the Larsen score, and 1.1 for the Lysholm score (Table 1). SRM > 0.8 is generally considered to be excellent.

#### Discriminant ability

Ceiling and floor effects, which also have an effect on the responsiveness of a measure, were absent.

## Discussion

The most important findings of the present study are the good psychometric properties, good reliability, and good construct validity of the Italian version of the Kujala, Fulkerson, Larsen, and Lysholm questionnaires. In addition, they all proved to be very useful when measuring changes in the symptoms of patients with patellofemoral pathologies over time in an objective manner. No difficulties were encountered in translating the questionnaires, and the back-translations corresponded very well to the original English versions.

The Cronbach alpha coefficient for the Kujala, Fulkerson, Larsen and Lysholm scores ranged between 0.091 and 0.930, indicating excellent internal consistency. These data are consistent with Cronbach alpha values obtained in translations of the same questionnaires into other languages [2, 7, 9].

The test–retest reliability was determined using the ICC with 5 days between each administration of the questionnaire. In our case, the ICC ranged between 0.92 and 0.96, revealing excellent short-term reliability. These values are similar to those of the original version and similar to those of the questionnaires translated into other languages [2, 7, 9].

Furthermore, the four scoring systems were correlated to the Oxford knee score, which served as gold standard. The Oxford questionnaire has been previously adapted to Italian culture and is a universally accepted tool to evaluate knee pathology. Pearson’s correlation was 0.96 between the Kujala and Oxford scores, 0.90 between the Larsen and Oxford scores, 0.94 between the Lysholm and Oxford scores, and 0.93 between the Fulkerson and Oxford scores. The Kujala questionnaire demonstrated the strongest correlation with the Oxford knee score as well as the best test–retest reliability. The Lysholm, Fulkerson and Larsen scores also exhibited high correlation and test–retest reliability.

The quality of measurement questionnaires has usually been evaluated by considering the reliability and validity of such questionnaires; it has, however, been suggested that responsiveness should be another criterion in the choice of a measurement questionnaire. Assessment of the sensitive to change showed that the Italian versions of the four questionnaires showed good sensitivity to change and may be useful in objectively assessing changes during the use of specific interventions (conservative or surgical) in the treatment of patellofemoral pathologies.

The questionnaires were easily understood by the participants, and it took them less than 10 min to complete the four of them independently. All patients fully completed the questionnaires, resulting in the maximum response rate. In addition, the lack of floor and ceiling effects confirm that this version of the aforementioned scoring scales are an appropriate tools for a broad spectrum of patients with a different severities patellofemoral pathologies.

Therefore the present study successfully translated and adapted four of the most common questionnaire for patellofemoral pathologies and to assess the efficacy of the proposed treatments. The present study was not without limitations. First of all, the statistical power of the study was not calculated; however, we referred to similar studies available in literature to determine the sample size needed. Secondly, our population included only patients affected by patellofemoral symptoms, thus limiting the characteristics of our population. Despite these limitations, we can conclude that the Italian versions of the Kujala, Larsen, Lysholm and Fulkerson scoring systems are equivalent to their English versions, given their reproducibility, consistency and validity.

Currently no Italian validated version of the Kujala, Larsen, Lysholm and Fulkerson scoring systems are available. The present study confirms that the aforementioned scoring systems have high test–retest reliability and high correlation with the Oxford knee score. In addition, the absence of ceiling and floor effects, confirms that they can be used in Italian patients with patellofemoral disorders.

## Additional file

**Additional file 1.** Italian version of Kujala, Larsen, Lysholm and Fulkerson scoring system.
