# Supplementary material for: Cross-cultural adaptation and validation of the Italian versions of the Kujala, Larsen, Lysholm and Fulkerson scores in patients with patellofemoral disorders
Source: J Orthop Traumatol. 2018 Sep 12;19(1):18. doi: 10.1186/s10195-018-0508-9 (PMC6135726; doi:10.1186/s10195-018-0508-9)
Supplement: Supplementary file 1 — Additional file 1. Italian version of Kujala, Larsen, Lysholm and Fulkerson scoring system. [file 10195_2018_508_MOESM1_ESM.docx]

**KUJALA SCORING SYSTEM**

Kujala UM, Jaakkola LH, Koskinen SK, Taimela S, Hurme M, Nelimarkka O. Scoring of patellofemoral disorders. Arthroscopy. 1993;9(2):159-163.

**Zoppia**

1 Nessuna 5 punti

2 Leggera o periodica 3 punti

3 Costante 0 punti

**Carico**

1 Carico completo senza dolore 5 punti

2 Doloroso 3 punti

3 Carico impossibile 0 punti

**Perimetro di marcia**

1 Illimitato 5 punti

2 Più di 2km 3 punti

3 1-2 km 2 punti

4 Impossibile 0 punti

**Scale**

1 Nessuna difficoltà 10 punti

2 Leggero dolore alla discesa delle scale 8 punti

3 Dolore sia alla salita che alla discesa 5 punti

4 Impossibile 0 punti

**Accovacciarsi**

1 Nessuna difficoltà 5 punti

2 Accovacciamenti ripetuti dolorosi 4 punti

3 Doloroso ogni volta 3 punti

4 Possibile ma con carico parziale 2 punti

5 Impossibile 0 punti

**Correre**

1 Nessuna difficoltà 10 punti

2 Dolore dopo più di 2 km 8 punti

3 Leggero dolore dall’inizio 6 punti

4 Dolore intenso 3 punti

5 Impossibile 0 punti

**Saltare**

1 Nessuna difficoltà 10 punti

2 Leggera difficoltà 7 punti

3 Dolore costante 2 punti

4 Impossibile 0 punti

**Posizione seduta prolungata a ginocchia flesse**

1 Nessuna difficoltà 10 punti

2 Dolore dopo esercizio 8 punti

3 Dolore costante 6 punti

4 Dolore all’estensione temporanea delle ginocchia 4 punti

5 Impossibile 0 punti

**Dolore**

1 Nessuno 10 punti

2 Leggero occasionale 8 punti

3 Tale da disturbare il riposo notturno 6 punti

4 Occasionalmente intenso 3 punti

5 Costantemente Intenso 0 punti

**Gonfiore**

1 Nessuno 10 punti

2 Dopo esercizi pesanti 8 punti

3 Dopo attività quotidiane 6 punti

4 Ogni sera 4 punti

5 Costante 0 punti

**Movimenti rotulei anomali dolorosi (sublussazioni)**

1 Nessuno 10 punti

2 Occasionali nelle attività sportive 6 punti

3 Occasionali nelle attività quotidiane 4 punti

4 Almeno una lussazione documentata 2 punti

5 Più di 2 lussazioni 0 punti

**Atrofia della coscia**

1 Nessuna 5 punti

2 Leggera 3 punti

3 Intensa 0 punti

**Deficit di flessione**

1 Nessuno 5 punti

2 Leggero 3 punti

3 Grave 0 punti

**LARSEN E LAURIDSEN SCORE CRITERIA**

Larsen E., Lauridsen F. Conservative treatment of patellar dislocations:influence of evident factors on the tendency to redislocation and therapeutic result. Clin Orthop. 1982;171:131-136

**Dolore**

a Nessuno 4

b Occasionale o minimo 3

c Dopo attività moderate 2

d Persistente 1

**Rigidità**

a Nessuno 4

b Occasionale o moderata 3

c Dopo attività moderate 2

d Persistente 1

**Crepitio retro-rotuleo**

a Nessuno 4

b Solo ai movimenti passivi 3

c Dopo attività moderate 2

d Persistente 1

**Limitazione della flessione**

a Nessuna 4

b 0°-5° 3

c 6°-10° 2

d > 10° 1

**Perdita di funzione**

a Nessuna 4

b Riduzione dell'attività sportiva strenua, ma non delle altre attività 3

c Moderata riduzione dell'attività sportiva e/o delle atre attività 2

d Completa riduzione dell'attività sportiva e/o delle altre attività 1

**LYSHOLM KNEE SCORING SCALE**

Tegner Y, LyshomJ: Rating systems in the evaluation of the knee ligament injuries. Clinical Orthop. AndRel Res, 1985,198:43-49

**Zoppia**

a Nessuna 5

b Leggera o episodica 3

c Grave e costante 0

**Ausilio alla deambulazione**

a Nessuno 5

b Bastone o canadese 2

c Carico impossibile 0

**Blocco articolare**

a Nessuna sensazione di blocco 15

b Sensazione di rigidità ma non di blocco 10

c Blocco occasionalmente 6

d Blocco frequentemente 2

e Blocco al momento dell'esame 0

**Instabilità**

a Nessuna instabilità

b Raramente durante l'attività sportiva o altre attività pesanti 25

c Frequentemente durante l'attività sportiva o altre attività pesanti 20

(oppure impossibilità alle partecipazioni) 15

d Occasionalmente in attività quotidiane 10

e Spesso in attività quotidiane 5

f Ad ogni passo 0

**Dolore**

a Nessuno 25

b Incostante e leggero durante esercizi pesanti 20

c Notevole durante l'esercizio pesante 15

d Notevole durante o dopo aver camminato più di 2 km 10

e Notevole durante o dopo aver camminato meno di 2 km 5

f Costante 0

**Gonfiore**

a Nessuno 10

b Durante un' attività pesante 6

c Durante un' attività abituale 2

d Costante 0

**Salire le scale**

a Nessun problema 10

b Qualche difficoltà 6

c Uno scalino alla volta 2

d Impossibile 0

**Accovacciarsi**

a Nessun problema 5

b Qualche difficoltà 4

c Non oltre 90° 2

d Impossibile 0

**FULKERSON SCORE**

Scala dell’instabilità modificata per la valutazione dell’instabilità e del dolore femoro-rotuleo

Fulkerson JP, Becker GJ, Meaney JA, Miranda M, Folcik MA. Anteromedial tibial tubercle transfer without bone graft. Am J Sports Med. 1990 Sep-Oct;18(5):490-496

**Zoppia**

Nessuna 10

Leggera 5

Grave 0

**Sostegni**

Completo 10

Bastone o stampelle talvolta necessari 3

Carico impossibile 0

**Scale**

Nessun problema 10

Leggera difficoltà 6

Un gradino alla volta 2

Impossibile 0

**Accovacciarsi**

Nessun problema 5

Leggera difficoltà 4

Non oltre i 90° di flessione 2

Impossibile 0

**Instabilità**

Mai presente 10

Con attività energiche 7

Occasionalmente nelle attività quotidiane 5

Frequentemente nelle attività quotidiane 3

Quotidianamente 0

**Dolore**

Nessuno 45

Leggero unicamente negli esercizi pesanti 40

Moderato negli esercizi pesanti 35

Intenso dopo esercizi pesanti 25

Intenso dopo aver camminato 2km 20

Intenso dopo aver camminato meno di 1km 10

Costante ed intenso 2

**Gonfiore**

Nessuno 10

Talvolta presente 7

A seguito di sforzi intensi 5

A seguito di sforzi moderati 2

Costante 0
